# Association between blood transfusion and early mortality in patient undergoing extracorporeal membrane oxygenation

**DOI:** 10.1038/s41598-025-11702-7

**Published:** 2025-07-25

**Authors:** Yonghoon Shin, Kwang-Sig Lee, Jinah Cha, Sunwoo Nam, Jun Ho Lee, Ji Eon Kim, Jae Seung Jung, Ho Sung Son, Ki Hoon Ahn, Hee Jung Kim

**Affiliations:** 1 Department of Cardiology, Cardiovascular Center, Korea University Anam Hospital, Korea University College of Medicine, Seoul, Republic of Korea; 2 AI Center, Korea University Anam Hospital, Korea University College of Medicine, Seoul, Republic of Korea; 3 Department of Biomedical Sciences, Korea University College of Medicine, Seoul, Republic of Korea; 4 Department of Obstetrics and Gynecology, Korea University Anam Hospital, Korea University College of Medicine, Seoul, Republic of Korea; 5 Department of Thoracic and Cardiovascular Surgery, Korea University Anam Hospital, Korea University College of Medicine, 73, Goryeodae-ro, Sungbuk-gu, Seoul, 02841 Republic of Korea

**Keywords:** Prognosis, Outcomes research, Risk factors, Cardiovascular diseases, Respiratory tract diseases

## Abstract

Blood transfusions are frequently administered to extracorporeal membrane oxygenation (ECMO) patients. However, the relationship between blood transfusion and outcomes in ECMO patients remains unclear. This study investigated the association between blood transfusion and 90-day mortality in ECMO patients. Using a large administrative database (National Health Insurance Service, NHIS) from the Republic of Korea during 2014–2020, ECMO cases were identified. Patients younger than 19 years and those with ECMO durations of less than 1 day were excluded. Transfusion variables included the total volumes of red blood cells (RBC), fresh frozen plasma (FFP), and platelet concentrates (PC), normalized by hospital stays. The primary outcome was 90-day mortality. Variable importance calculation using Random Forest and XGBoost models identified RBC, FFP, and PC transfusions among the top 10 predictors of mortality. Variable importance was calculated to identify the most influential variables in predicting the outcome among 51 variables. A total of 11,874 patients were included. The median age was 62 years (IQR 51–71), and 32.7% were female. 90-day mortality occurred in 6980 patients (58.8%). The proportion of patients who received at least one transfusion during hospitalization was 96.2% for RBC, 68.8% for FFP, and 74.4% for PC. Higher transfusion volumes (50–100%) were consistently associated with an increased risk of death compared to lower volumes (0–49%). Odds ratios for mortality were 6.764 (95% confidence interval (CI), 6.227–7.347) for RBC/hospital day, 3.702 (95% CI 3.426–4.000) for FFP/hospital day, and 5.082 (95% CI 4.691–5.506) for PC/hospital day in the higher transfusion group. In a multivariable logistic regression model, RBC, FFP, and PC transfusions normalized by hospital stay were significantly associated with mortality (*p* < 0.001). Variable importance calculation using Random Forest and XGBoost models identified RBC, FFP, and PC transfusions among the top 10 predictors of mortality. Blood transfusion normalized by hospital stay emerged as a critical predictor of 90-day mortality in ECMO patients. Thus, optimizing blood transfusion practices is essential to improve patient outcomes and safety.

**Supplementary Information:**

The online version contains supplementary material available at 10.1038/s41598-025-11702-7.

## Introduction

Extracorporeal membrane oxygenation (ECMO) has emerged as a crucial tool in the management of acute cardiac or respiratory failure refractory to conventional therapy^1^. Recently, the number of ECMO cases has increased rapidly^2^. Notably, blood transfusion is administered to most ECMO patients, and the transfusion frequency exceeds rates observed in general critical care populations^3–5^. ECMO patients require more transfusions due to hemodilution, hemolysis, coagulopathy, and anticoagulation needs^6^. Another limitation of our approach is the normalization of tr Despite the common practice of blood transfusions in ECMO patients, optimal transfusion strategies remain debated. While ELSO guidelines acknowledge that ECMO patients may require different transfusion approaches than typical critically ill patients, they do not provide specific hemoglobin thresholds due to limited evidence^7,8^. Previous studies have demonstrated varying outcomes based on transfusion thresholds, and thus the impact of transfusion in ECMO patients remains unclear^9,10^. Therefore, this study aimed to investigate the association between blood transfusion and 90-day mortality in ECMO patients using nationwide real-world data, analyzing predictive factors for 90-day mortality and evaluating the importance of blood transfusion through machine learning analysis.

## Methods

### Analytic workflows

**Fig. 1:**
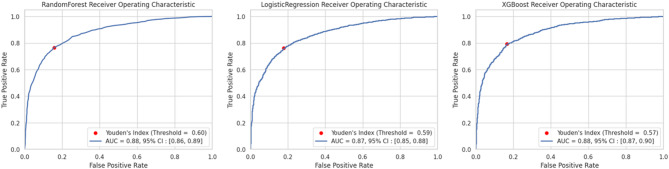
Analytic workflows.

This study employed a comprehensive six-step analytical approach to develop robust predictive models for 90-day mortality in ECMO patients (Fig. 1). We began with data acquisition from the Korean National Health Insurance Service (NHIS) database, encompassing 11,874 ECMO patients treated between 2014 and 2020 with a minimum follow-up period of 1 year. Subsequently, we conducted systematic variable selection, identifying 51 clinical variables including patient demographics, comorbidities, procedural interventions, transfusion data, and ECMO duration for inclusion in our predictive models. The modeling phase involved development of three distinct predictive algorithms: Logistic Regression, Random Forest, and XGBoost, each offering unique advantages in handling complex clinical data relationships. Model performance was rigorously evaluated through multiple complementary metrics including area under the curve (AUC), accuracy, sensitivity, specificity, Cohen’s Kappa coefficient, and confusion matrix analysis to ensure comprehensive assessment of predictive capabilities. To enhance clinical interpretability and identify key predictors, we conducted detailed interpretability assessments using variable importance calculations, SHAP (Shapley Additive Explanations) values, and systematic review of the top 10 predictors across all models. Finally, all analyses were implemented using Python 3.8.16 with specialized packages including scikit-learn for machine learning algorithms, XGBoost for gradient boosting, SHAP for model interpretation, and matplotlib and seaborn for data visualization, ensuring reproducible and methodologically sound results.

### Dataset

The dataset for this study was sourced from the National Health Insurance Service (NHIS) database, a comprehensive healthcare system in the Republic of Korea covering about 98% of the population^11–13^. The NHIS database collects anonymized medical claims data from healthcare providers, which is widely utilized for health services research. All diagnoses were recorded using ICD codes, and procedures covered by insurance were documented. Based on these records, data on comorbidities, ECMO-related diagnoses, concomitant procedures, transfusion characteristics, and mortality were collected and analyzed. Patients who underwent ECMO from 2014 to 2020 were identified from the NHIS database. The last follow-up date was December 2021, ensuring a follow-up period of at least 1 year for all subjects. Exclusion criteria included patients younger than 19 years and those whose ECMO duration was less than 1 day (indicating failure to maintain ECMO). Of the 14,398 ECMO cases identified, 724 patients were excluded due to age under 19 years, and 1797 were excluded due to ECMO being discontinued within 1 day, resulting in a final sample of 11,874 patients. This study was approved by the Institutional Review Board (IRB) of Korea University Anam Hospital (Approval No.2023AN0271: Approve date, 29/June/2029). As the research utilized anonymized patient data, the requirement for informed consent was waived by the IRB.

To account for regional and institutional variability in care, two variables were used. The first variable, capital area, was defined as whether the ECMO procedure was performed in a hospital located within the Seoul Metropolitan Area, which includes Seoul, Incheon, and Gyeonggi regions with the highest concentration of tertiary hospitals and medical resources in Korea. The second variable, ECMO center volume, was represented by annual ECMO case volume per institution, categorized into quartiles (Q1–Q4). Q1 corresponds to the lowest volume centers a year, while Q4 represents the highest volume centers.

### Predictive modeling for 90-day mortality using random forest, logistic regression, and XGBoost

**Fig. 2:**
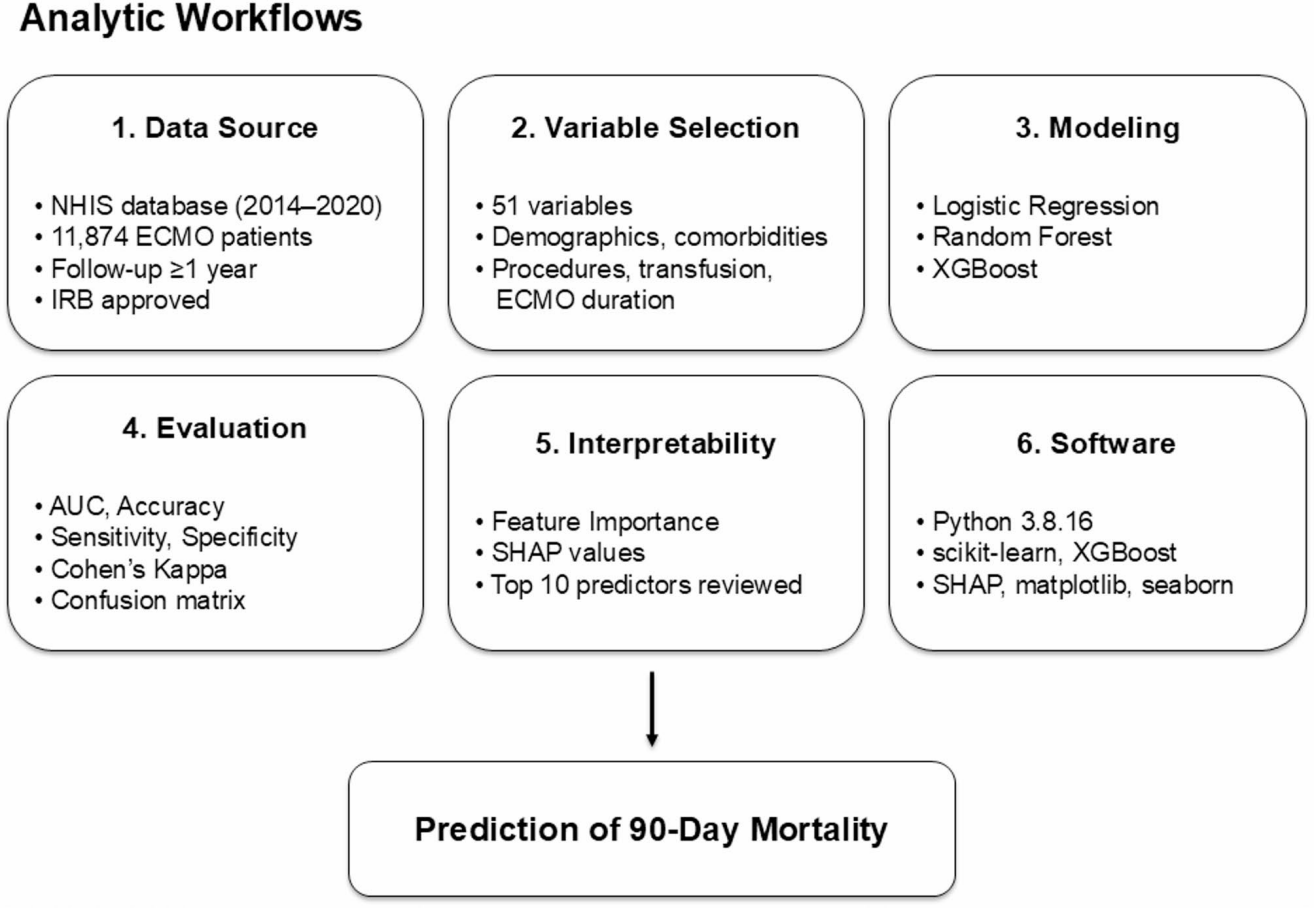
Glossary of machine learning terms.

To facilitate understanding for readers across different medical specialties, detailed definitions of key machine learning terminology are provided in a separate glossary (Fig. 2). Three predictive models—random forest, logistic regression, and XGBoost—were employed to predict 90-day mortality among patients treated with ECMO. A random forest is a group of decision trees which make majority votes on the dependent variable (“bootstrap aggregation”)^14–17^. In this study, we implemented a random forest with 1000 decision trees, where each tree was trained on a bootstrapped sample of the training data. Logistic regression, a traditional and interpretable method, was used as a baseline model for estimating the relationship between predictors and 90-day mortality. XGBoost, a gradient boosting algorithm, was included to optimize predictive accuracy through iterative learning, with hyperparameters such as the learning rate and tree depth tuned on the training set to enhance model performance. The dataset was randomly split into training (80%) and testing (20%) subsets. Model performance was primarily evaluated using the area under the receiver-operating-characteristic curve (AUC) (area under the plot of sensitivity vs. 1—specificity), along with sensitivity, specificity, accuracy, and Cohen’s Kappa. These metrics provided a robust understanding of the models’ predictive capabilities, while the confusion matrix offered detailed insights into the distribution of correct and incorrect predictions, highlighting the models’ strengths and weaknesses. This study was conducted and reported in accordance with the Strengthening the Reporting of Observational Studies in Epidemiology (STROBE) guidelines for observational studies and the Transparent Reporting of a multivariable prediction model for Individual Prognosis Or Diagnosis (TRIPOD) statement for the predictive modeling components. All analyses were performed using Python 3.8.16. We used scikit-learn (v1.3.2) for model development and evaluation, xgboost (v0.8.0) for gradient boosting, statsmodels (v0.14.0) for statistical analysis, shap (v0.32.0) for model interpretation, pandas (v1.4.4) and numpy (v1.19.5) for data preprocessing and handling, and matplotlib (v3.6.3) and seaborn (v0.12.2) for data visualization.

### Variables

A total of 51 variables were analyzed in this study, with a wide range of demographic, therapeutic, clinical, and temporal factors to evaluate their association with 90-day mortality in ECMO patients. Demographic variables included age, sex (Female), residence in the capital area, and economic variable, providing insights into patient characteristics. Therapeutic factors focused significantly on transfusion information, including the volume of RBC, FFP, and PC transfused during hospitalization to normalize transfusion usage against hospitalization duration. Additionally, ECMO duration and major procedures such as CABG and PCI were considered as part of the therapeutic profile. Cardiovascular conditions, such as ischemic heart disease (IHD), myocardial infarction, cardiogenic shock, and heart failure, were included alongside surgical interventions like valve surgery and aortic surgery. Other medical conditions, including chronic renal failure (CRF), cancer, diabetes (DM), pulmonary disease, COVID-19, myocarditis, and pulmonary embolism, were also evaluated to provide a comprehensive understanding of factors influencing patient outcomes.

### Variable importance calculation methods

To assess variable importance and ensure interpretability, we employed specific variable importance calculation methods tailored to each model. Random Forest was utilized to identify key predictors of mortality and to evaluate the strength of their associations, with variable importance calculated using impurity-based measures, which quantify the reduction in node impurity contributed by each predictor across all trees^18–21^. This approach identifies the predictors that most effectively split the data at decision nodes, with key variables emerging as important determinants of 90-day mortality in ECMO patients. For Logistic Regression, variable importance was derived from the absolute values of model coefficients, which represent both the strength and the direction of each predictor’s association with the outcome. In addition, for XGBoost, variable importance was calculated using the variable importances attribute, which ranks variable by their contribution to predictive accuracy through gradient-based optimization. To gain deeper insights into the primary factors associated with 90-day mortality, we analyzed the top 10 variable with the highest importance scores across all models. This multi-model approach enabled a comprehensive evaluation of variable importance, facilitating a robust and interpretable framework for identifying predictors of mortality. Such an approach ensures not only the clinical relevance of the findings but also enhances the transparency and trustworthiness of the predictive models. Finally, it can be noted that Python (version 3.8.16) was employed for the analysis conducted in this study.

## Results

### Baseline characteristics

Among 11,874 patients included in the analysis, the median age was 62 years (interquartile range [IQR]: 51–71), with female patients comprising 32.7% of the cohort (Table 1). The study population showed significant differences between survivors (n = 4894) and non-survivors (n = 6980), with median age (58 vs. 64.5 years, *p* < 0.001) being notably different. Regarding comorbidities, hypertension (58.0% vs. 67.8%, *p* < 0.001), dyslipidemia (63.2% vs. 69.0%, *p* < 0.001), and diabetes mellitus (43.7% vs. 50.6%, *p* < 0.001) were most prevalent. Cardiovascular comorbidities included previous myocardial infarction (20.0% vs. 22.8%, *p* < 0.001), congestive heart failure (10.3% vs. 12.3%, *p* = 0.001), and ischemic stroke (9.3% vs. 12.8%, *p* < 0.001). Notable differences were also observed in the prevalence of chronic renal failure (11.3% vs. 18.4%, *p* < 0.001) and cancer history within 5 years (15.8% vs. 20.5%, *p* < 0.001). The primary diagnoses at ECMO initiation included ischemic heart disease (43.3% vs. 42.8%, *p* = 0.602), pulmonary disease (43.0% vs. 42.0%, *p* = 0.304), and cardiac arrest (21.1% vs. 21.9%, *p* = 0.296). The median duration of ECMO support was 5 days, with significant differences in hospital length of stay (30 vs. 13 days, *p* < 0.001) and intensive care unit stay (24 vs. 15 days, *p* < 0.001). Concomitant procedures included percutaneous coronary intervention (27.7% vs. 27.5%, *p* = 0.792), coronary artery bypass grafting (10.6% vs. 9.3%, *p* = 0.023), and valve surgery (7.3% vs. 9.6%, *p* < 0.001).

**Table 1:** Baseline characteristics.

Variables	Total cohort (N = 11,874)	Survivor (N = 4894)	Death (N = 6980)	*p*
Female n (%)	3881 (32.68)	1555 (31.77)	2326 (33.32)	0.080
Age, (range)	62 (51–71)	58 (48–67)	64.5 (55–73)	< 0.001
Economic status, (range)	12 (6–17)	12 (6–17)	12 (6–17)	0.618
Capital area	5403 (45.5)	2279 (46.57)	3124 (44.76)	0.053
Year of ECMO				
2014–2015, n (%)	2379 (20.04)	861 (17.59)	1518 (21.75)	< 0.001
2016–2017, n (%)	3091 (26.03)	1259 (25.73)	1832 (26.25)	0.538
2018–2019, n (%)	4095 (34.49)	1744 (35.64)	2351 (33.68)	0.029
2020, n (%)	2309 (19.45)	1030 (21.05)	1279 (18.32)	< 0.001
Hospital cases by annual case per year				
Q1, n (%)	157 (1.32)	43 (0.88)	114 (1.63)	0.001
Q2, n (%)	1142 (9.62)	369 (7.54)	773 (11.07)	< 0.001
Q3, n (%)	2589 (21.8)	913 (18.66)	1676 (24.01)	< 0.001
Q4, n (%)	7986 (67.26)	3569 (72.93)	4417 (63.28)	< 0.001
Comorbidity				
Dyslipidemia, n (%)	7907 (66.59)	3091 (63.16)	4816 (69)	< 0.001
Hypertension, n (%)	7571 (63.76)	2837 (57.97)	4734 (67.82)	< 0.001
Diabetes Mellitus, n (%)	5671 (47.76)	2138 (43.69)	3533 (50.62)	< 0.001
Myocardial Infarction, n (%)	2565 (21.6)	977 (19.96)	1588 (22.75)	< 0.001
Cancer, n (%)	2202 (18.54)	773 (15.79)	1429 (20.47)	< 0.001
Chronic Kidney Disease, n (%)	1838 (15.48)	552 (11.28)	1286 (18.42)	< 0.001
Congestive Heart Failure, n (%)	1363 (11.48)	504 (10.3)	859 (12.31)	0.001
Ischemic stroke, n (%)	1352 (11.39)	456 (9.32)	896 (12.84)	< 0.001
COVID-19, n (%)	109 (0.92)	51 (1.04)	58 (0.83)	0.276
Charlson Comorbidity Index, (IQR)	4 (2–7)	5 (3–8)	3 (0–5)	< 0.001
Diagnosis categories at ECMO				
IHD + coronary procedure	7942 (66.9)	3245 (66.3)	4697 (67.3)	0.078
Pulmonary disease, n (%)	5035 (42.4)	2103 (42.97)	2932 (42.01)	0.304
Heart failure, n (%)	2606 (21.95)	1064 (21.74)	1542 (22.09)	0.666
Cardiac arrest, n (%)	2561 (21.57)	1032 (21.09)	1529 (21.91)	0.296
Cardiogenic shock, n (%)	2110 (17.77)	722 (14.75)	1388 (19.89)	< 0.001
Cardiomyopathy, n (%)	1049 (8.83)	522 (10.67)	527 (7.55)	< 0.001
Pulmonary thromboembolism, n (%)	552 (4.65)	273 (5.58)	279 (4)	< 0.001
Myocarditis, n (%)	428 (3.6)	257 (5.25)	171 (2.45)	< 0.001
Not categorized, n (%)	1072 (9.03)	402 (8.21)	670 (9.6)	0.010
IHD diagnosis				
Ischemic Heart Disease Diagnosis, n (%)	5104 (42.98)	2118 (43.28)	2986 (42.78)	0.602
ST-Elevation Myocardial Infarction, n (%)	1374 (11.57)	580 (11.85)	794 (11.38)	0.442
Non-ST-Elevation Myocardial Infarction, n (%)	1157 (9.74)	442 (9.03)	715 (10.24)	0.031
Angina, n (%)	2604 (21.93)	1115 (22.78)	1489 (21.33)	0.063
CPR at ER, n (%)	1795 (15.12)	692 (14.14)	1103 (15.8)	0.014
Concomitant procedure				
Percutaneous Coronary Intervention, n (%)	3271 (27.55)	1355 (27.69)	1916 (27.45)	0.792
Coronary Artery Bypass Grafting, n (%)	1165 (9.81)	517 (10.56)	648 (9.28)	0.023
Valve surgery, n (%)	1024 (8.62)	356 (7.27)	668 (9.57)	< 0.001
Aorta surgery, n (%)	517 (4.35)	165 (3.37)	352 (5.04)	< 0.001
Ventricular assist device, n (%)	35 (0.29)	27 (0.55)	8 (0.11)	< 0.001
Organ transplantation				
Lung Transplantation, n (%)	373 (3.14)	321 (6.56)	52 (0.74)	< 0.001
Heart Transplantation, n (%)	382 (3.22)	316 (6.46)	66 (0.95)	< 0.001
Heart Lung Transplantation, n (%)	7 (0.06)	5 (0.1)	2 (0.03)	0.215
Hospital progress				
ECMO operation day, (IQR)	5 (3–9)	5 (3–9)	5 (2–10)	0.004
Hospital stay, (IQR)	19 (9–31)	30 (18–42)	13 (6–25)	< 0.001
ICU stay, (IQR)	19 (8–38)	24 (12–45)	15 (6–33)	< 0.001
Ventilator day, (IQR)	9 (4–18)	10 (5–18)	8 (4–17)	< 0.001

IHD + coronary procedure: Includes patients diagnosed with IHD or those not diagnosed with IHD but who underwent PCI or CABG. Not categorized: diagnosis code was not included in IHD, Cardiogenic shock, Cardiac arrest, Pulmonary embolism, pulmonary disease. Cancer: Previous history of malignancy diagnosed within 5 years preceding hospital admission.

*IQR* Interquartile range, *CPR* Cardiopulmonary resuscitation, *ECMO* Extracorporeal membrane oxygenation, *ICU* Intensive care unit, *ER* Emergency room, *Q* Quartile.

### Blood transfusion profile

Blood product transfusion was common in the study population, with 96.2% of patients receiving red blood cells (RBCs) (Table 2). Fresh frozen plasma (FFP) and platelet concentrate (PC) were administered to 68.8% and 74.4% of patients, respectively. The median number of RBC units transfused during hospitalization was significantly lower in survivors compared to non-survivors (10 vs. 14 units, *p* < 0.001). A similar pattern emerged for FFP (3 vs. 6 units, *p* < 0.001) and PC (8 vs. 18 units, *p* < 0.001) requirements between groups. After normalization for hospital length of stay, the daily transfusion rates remained consistently lower in survivors for all blood products, with RBC (0.35 vs. 1.0 units/day, *p* < 0.001), FFP (0.08 vs. 0.44 units/day, *p* < 0.001), and PC (0.23 vs. 1.45 units/day, *p* < 0.001). The proportion of patients receiving transfusions was also lower in survivors across all blood components, with RBC administration in 95.3% vs. 96.9% (*p* < 0.001), FFP in 60.6% vs. 74.6% (*p* < 0.001), and PC in 65.8% vs. 80.4% (*p* < 0.001) of patients.

**Table 2:** Blood transfusion profile during hospital stay.

Variables	Total cohort (N = 11,874)	Survivor (N = 4894)	Death (N = 6980)	*p*
RBC transfusion during Hospital stay, n (%)	11,422 (96.19)	4662 (95.26)	6760 (96.85)	< 0.001
Number of RBC transfusion during Hospital stay, (range)	12 (6–23)	10 (5–20)	14 (7–26)	< 0.001
RBC/hospital stay, (range)	0.63 (0.29–1.38)	0.35 (0.18–0.62)	1 (0.5–2)	< 0.001
FFP transfusion during Hospital stay, n (%)	8173 (68.83)	2965 (60.58)	5208 (74.61)	< 0.001
Number of FFP transfusion during Hospital stay, (range)	4 (0–13)	3 (0–9)	6 (0–16)	< 0.001
FFP/hospital stay, (range)	0.21 (0–0.79)	0.08 (0–0.28)	0.44 (0–1.38)	< 0.001
PC transfusion during Hospital stay, n (%)	8829 (74.36)	3218 (65.75)	5611 (80.39)	< 0.001
Number of PC transfusion during Hospital stay, (range)	13 (0–36)	8 (0–24)	18 (5–45)	< 0.001
PC/hospital stay, (range)	0.67 (0–2.19)	0.23 (0–0.74)	1.45 (0.29–3.4)	< 0.001

RBC/hospital stay, FFP/hospital stays, PC/hospital stays: The transfusion volumes of RBCs, FFP, and PC were normalized based on the length of hospital stay.

*RBC* Red blood cell, *FFP* Fresh frozen plasma, *PC* Platelet concentrate; range: median (25–75 interquartile).

### Association between transfusion and mortality

Univariable logistic regression analysis of transfusion volumes demonstrated significant associations with mortality risk (Table 3). When stratified by transfusion intensity, patients receiving higher transfusion volumes (50-100th percentile) showed markedly increased mortality compared to those receiving lower volumes (0-49th percentile). The mortality risk was most pronounced for RBC transfusion, with patients in the higher transfusion group showing significantly elevated odds compared to the lower transfusion group (OR 6.764 vs. 0.163, *p* < 0.001). Similar patterns were observed for PC (OR 5.082 vs. 0.284, *p* < 0.001) and FFP (OR 3.702 vs. 0.270, *p* < 0.001) transfusions. The incidence of death was consistently higher in patients receiving greater transfusion volumes, with rates of 5.611 vs. 2.553 for RBC, 5.430 vs. 2.515 for PC, and 5.183 vs. 2.542 for FFP in the higher versus lower transfusion groups, respectively. In the univariable analysis, each additional unit of blood product transfused was associated with increased mortality: RBC (OR 1.016, 95% CI 1.013–1.018, *p* < 0.001), PC (OR 1.011, 95% CI 1.009–1.012, *p* < 0.001), and FFP (OR 1.017, 95% CI 1.014–1.019, *p* < 0.001).

**Table 3:** Risk of the death according to the transfusion volume (univariable logistic regression).

	Total	Survivor	Non-survivor	IR of death	OR (95% CI)	*p*
RBC	11,422	4662	6760	4.143	1.016 (1.013–1.018)	< 0.001
RBC/hospital days (0 ~ 49%)	5484	3484	2000	2.553	0.163 (0.15–0.176)	< 0.001
RBC/hospital days (50 ~ 100%)	5938	1178	4760	5.611	6.764 (6.227–7.347)	< 0.001
FFP	8173	2965	5208	4.461	1.017 (1.014–1.019)	< 0.001
FFP/hospital days (0–49%)	2236	1424	812	2.542	0.27 (0.25–0.292)	< 0.001
FFP/hospital days (50–100%)	5937	1541	4396	5.183	3.702 (3.426–4)	< 0.001
PC	8828	3217	5611	4.449	1.011 (1.009–1.012)	< 0.001
PC/hospital days (0–49%)	2970	1903	1067	2.515	0.284 (0.26–0.309)	< 0.001
PC/hospital days (50–100%)	5858	1314	4544	5.43	5.082 (4.691–5.506)	< 0.001

RBC/hospital stay, FFP/hospital stays, PC/hospital stays: The transfusion volumes of RBCs, FFP, and PC were normalized based on the length of hospital stay.

*FFP* Fresh frozen plasma, *IR* Incidence rate, *OR* Odds ratio, *PC* Platelet concentrate, *RBC* Red blood cell.

Multivariable logistic regression analysis confirmed that transfusion volumes normalized by hospital stay remained independently associated with mortality after adjusting for potential confounders (Table 4). Higher normalized RBC transfusion volume maintained a strong association with mortality (OR 3.031, 95% CI 2.641–3.480, *p* < 0.001), as did PC (OR 1.481, 95% CI 1.371–1.598, *p* < 0.001) and FFP (OR 1.294, 95% CI 1.124–1.488, *p* < 0.001) transfusion volumes. Other significant predictors of mortality included age (OR 1.038, 95% CI 1.033–1.042, *p* < 0.001), chronic renal failure (OR 2.443, 95% CI 2.089–2.857, *p* < 0.001), and cancer history (OR 2.290, 95% CI 1.989–2.637, *p* < 0.001). Several cardiovascular conditions showed significant associations with mortality, including non-ST-elevation myocardial infarction (OR 1.307, 95% CI 1.047–1.633, *p* = 0.018), heart failure (OR 1.202, 95% CI 1.060–1.364, *p* = 0.004), and cardiogenic shock (OR 1.224, 95% CI 1.067–1.404, *p* = 0.004). Notably, certain interventions demonstrated protective effects, including coronary artery bypass grafting (OR 0.546, 95% CI 0.435–0.686, *p* < 0.001), lung transplantation (OR 0.180, 95% CI 0.125–0.260, *p* < 0.001), and heart transplantation (OR 0.150, 95% CI 0.104–0.216, *p* < 0.001).

**Table 4:** Univariable and multivariable analysis of logistic regression for the 90-day death.

Variables	Univariable		Multivariable	
	OR (95% CI)	*p*	OR (95% CI)	*p*
Female	1.073 (0.993–1.16)	0.076		
Age	1.029 (1.026–1.032)	< 0.001	1.038 (1.033–1.042)	< 0.001
Economic status	1.001 (0.995–1.007)	0.727	0.989 (0.981–0.996)	0.003
Capital area	0.93 (0.864–1)	0.051	0.88 (0.796–0.973)	0.013
Year of ECMO				
2014–2015	1.302 (1.186–1.429)	< 0.001		
2016–2017	1.027 (0.945–1.117)	0.524		
2018–2019	0.917 (0.85–0.99)	0.028		
2020	0.842 (0.768–0.922)	< 0.001		
Hospital cases				
Q1	1.873 (1.316–2.665)	< 0.001		
Q2	1.527 (1.341–1.739)	< 0.001		
Q3	1.378 (1.259–1.508)	< 0.001		
Q4	0.64 (0.591–0.693)	< 0.001		
Hypertension	1.528 (1.417–1.649)	< 0.001	1.129 (0.996–1.279)	0.057
Diabetes Mellitus	1.321 (1.228–1.422)	< 0.001	1.542 (1.372–1.733)	< 0.001
Dyslipidemia	1.298 (1.202–1.402)	< 0.001		
Ischemic stroke	1.433 (1.272–1.615)	< 0.001	1.256 (1.064–1.483)	0.007
Myocardial Infarction	1.181 (1.079–1.292)	< 0.001		
Congestive Heart Failure	1.222 (1.088–1.374)	0.001		
Cancer	1.372 (1.246–1.511)	< 0.001	2.29 (1.989–2.637)	< 0.001
Chronic Kidney Disease	1.777 (1.596–1.978)	< 0.001	2.443 (2.089–2.857)	< 0.001
COVID19	0.796 (0.545–1.161)	0.236	0.636 (0.398–1.017)	0.059
Charlson Comorbidity Index	0.836 (0.826–0.845)	< 0.001	0.708 (0.694–0.722)	< 0.001
Diagnosis at ECMO				
CPR at ER	1.14 (1.028–1.263)	0.013		
Ischemic Heart Disease	0.98 (0.91–1.055)	0.589	1.306 (0.999–1.708)	0.051
ST-Elevation Myocardial Infarction	0.955 (0.852–1.07)	0.425	1.177 (0.918–1.509)	0.198
Non-ST-Elevation Myocardial Infarction	1.15 (1.015–1.302)	0.028	1.307 (1.047–1.633)	0.018
Angina	0.919 (0.842–1.004)	0.06		
IHD + coronary procedure	1.045 (0.967–1.13)	0.261	1.281 (0.939–1.746)	0.118
Percutaneous Coronary Intervention	0.988 (0.911–1.072)	0.776	0.774 (0.628–0.953)	0.016
Coronary Artery Bypass Grafting	0.866 (0.767–0.979)	0.021	0.546 (0.435–0.686)	< 0.001
Myocarditis	0.453 (0.372–0.552)	< 0.001	0.493 (0.376–0.647)	< 0.001
Pulmonary thromboembolism	0.705 (0.594–0.836)	< 0.001	0.542 (0.426–0.689)	< 0.001
Cardiomyopathy	0.684 (0.602–0.777)	< 0.001		
Heart failure	1.021 (0.934–1.115)	0.649	1.202 (1.06–1.364)	0.004
Cardiogenic shock	1.434 (1.3–1.583)	< 0.001	1.224 (1.067–1.404)	0.004
Cardiac arrest	1.05 (0.96–1.147)	0.286		
Pulmonary disease	0.961 (0.893–1.035)	0.295	1.185 (1.048–1.341)	0.007
Not categorized	1.186 (1.042–1.35)	0.01	1.174 (0.946–1.457)	0.145
Valve surgery	1.349 (1.18–1.543)	< 0.001	0.577 (0.466–0.715)	< 0.001
Aorta surgery	1.522 (1.26–1.838)	< 0.001	0.771 (0.563–1.054)	0.103
Lung transplantation	0.107 (0.08–0.144)	< 0.001		
Heart transplantation	0.138 (0.106–0.181)	< 0.001		
Heart–lung transplantation	0.28 (0.054–1.445)	0.129		
Ventricular assist device	0.207 (0.094–0.456)	< 0.001	0.271 (0.105–0.7)	0.007
ECMO operation day	1.007 (1.002–1.011)	0.004	1.031 (1.024–1.038)	< 0.001
Number of RBC transfusion during Hospital stay	1.016 (1.013–1.018)	< 0.001	0.969 (0.963–0.975)	< 0.001
RBC/hospital stay	3.835 (3.565–4.125)	< 0.001	3.031 (2.641–3.48)	< 0.001
Number of FFP transfusion during Hospital stay	1.017 (1.014–1.019)	< 0.001		
FFP/hospital stay	3.202 (2.962–3.461)	< 0.001	1.294 (1.124–1.488)	< 0.001
Number of PC transfusion during Hospital stay	1.011 (1.009–1.012)	< 0.001		
PC/hospital stay	1.924 (1.855–1.996)	< 0.001	1.481 (1.371–1.598)	< 0.001

IHD + coronary procedure: Includes patients diagnosed with IHD or those not diagnosed with IHD but who underwent PCI or CABG. Not categorized: diagnosis code was not included in IHD, Cardiogenic shock, Cardiac arrest, Pulmonary embolism, pulmonary disease. RBC/hospital stay, FFP/hospital stays, PC/hospital stays: The transfusion volumes of RBCs, FFP, and PC were normalized based on the length of hospital stay. Cancer: Previous history of malignancy diagnosed within 5 years preceding hospital admission.

*CI* Confidence interval, *CPR* Cardiopulmonary resuscitation, *ECMO* Extracorporeal membrane oxygenation, *ER* Emergency room, *FFP* Fresh frozen plasma, *OR* Odds ratio, *PC* Platelet concentrate, *Q* Quartile, *RBC* Red blood cell.

To examine the relationship between transfusion and mortality in specific clinical contexts, we conducted subgroup analyses comparing transfusion-mortality relationships between patients with ischemic heart disease undergoing coronary procedures versus those undergoing cardiac valve or aortic surgery (Supplementary tables 1 and 2). The ischemic heart disease with coronary procedure group included 2110 patients, while the cardiac valve or aortic surgery group comprised 1024 patients. Both subgroups demonstrated significant associations between transfusion variables and mortality. In the ischemic heart disease with coronary procedure group, RBC/hospital stay showed an OR of 3.43 (95% CI 2.57–4.57, *p* < 0.001), while the cardiac valve or aortic surgery group demonstrated an OR of 1.85 (95% CI 1.41–2.43, *p* < 0.001). Similarly, FFP and PC transfusions normalized by hospital stay remained significantly associated with mortality in both subgroups (all *p* < 0.05).

### Machine learning-based mortality prediction

**Fig. 3:**
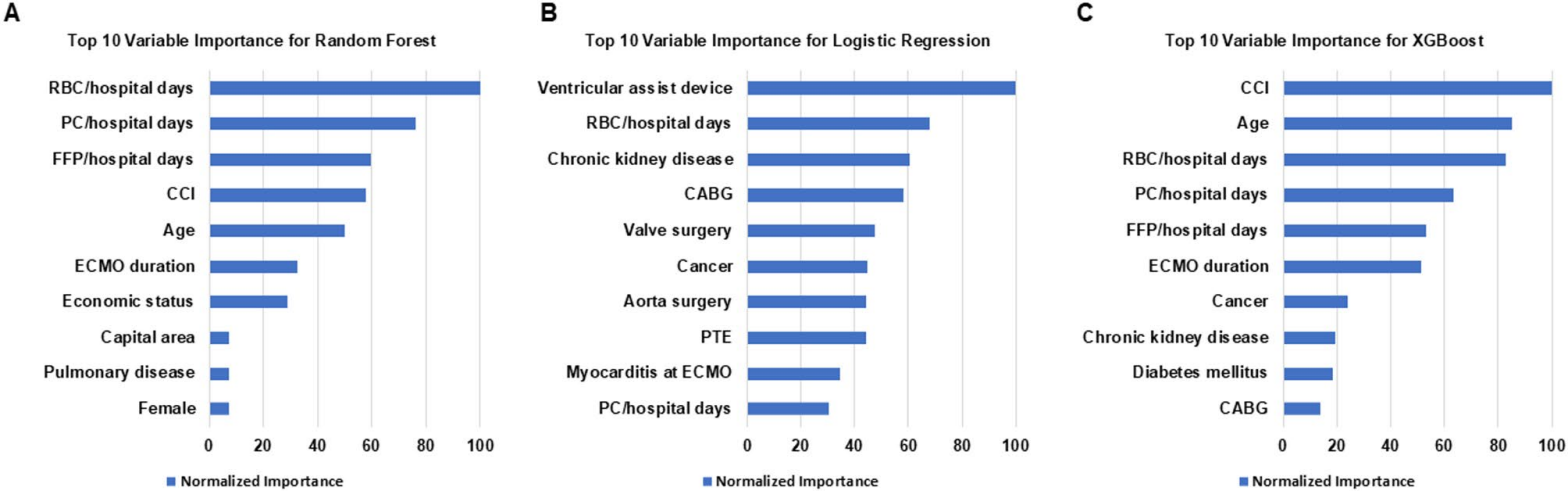
Top 10 variable importances from the machine learning analysis. (**A**) Top 10 Variable Importances (Normalized) for Random Forest. (**B**) Top 10 Variable Importances (Normalized) for Logistic Regression. (**C**) Top 10 Variable Importances (Normalized) for XGBoost. *RBC* Red blood cell, *FFP* Fresh frozen plasma, *PC* Platelet concentrate, *ECMO* Extracorporeal membrane oxygenation, *CCI* Charlson comorbidity index.

**Fig. 4:**
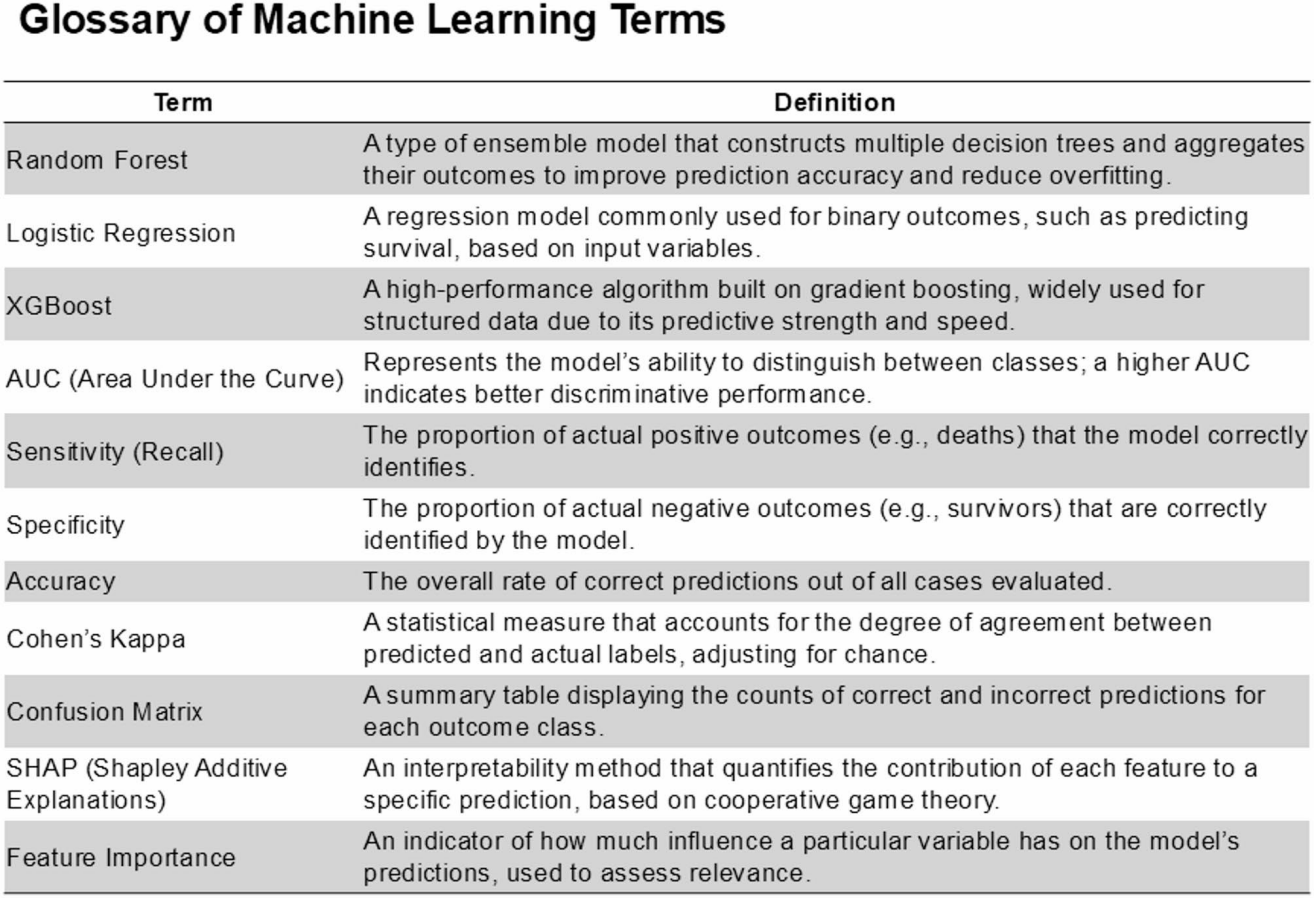
Receiver operating characteristic (ROC) curves for RandomForest, Logistic Regression, and XGBoost Models.

Three machine learning models were developed to predict 90-day mortality, with XGBoost demonstrating the highest predictive performance (AUC 0.909), followed by Random Forest (AUC 0.900) and logistic regression (AUC 0.891) (Table 5). XGBoost achieved the highest sensitivity (0.857) and specificity (0.793), with an accuracy of 0.830 and kappa coefficient of 0.651. Variable importance analysis revealed distinct patterns across models, though blood product transfusion metrics consistently ranked among the top predictors in Random Forest and XGBoost models (Fig. 3 and supplementary table 3). The Random Forest model identified RBC/hospital days as the primary predictor (importance score 0.135), followed by PC/hospital days (0.101) and FFP/hospital days (0.098). The XGBoost model prioritized the Charlson Comorbidity Index (importance score 0.169), RBC/hospital days (0.119), and absolute RBC volume (0.106) as the three most influential variable. Age and ECMO duration appeared among the top 10 predictors in both Random Forest and XGBoost analyses. The ROC curves demonstrated comparable discriminative ability among the three models, with XGBoost showing marginally superior performance across all sensitivity and specificity thresholds (Fig. 4). All models maintained high predictive accuracy, with kappa coefficients ranging from 0.622 to 0.651, indicating substantial agreement between predicted and observed outcomes.

**Table 5:** Machine learning performance.

Model	Sensitivity (recall)	Specificity	Kappa	Accuracy	ROC-AUC
Logistic Regression	0.843	0.779	0.622	0.816	0.891
Random Forest	0.842	0.785	0.626	0.818	0.900
XGBoost	0.857	0.793	0.651	0.83	0.909

## Discussion

This study demonstrates that blood transfusion, normalized by hospital stay, is a strong predictor of 90-day mortality in ECMO patients. Our main findings are as follows. First, blood product transfusion was nearly universal in ECMO patients, with RBC transfusion rates exceeding 96%. Second, higher normalized transfusion volumes were significantly associated with increased mortality, with odds ratios of 6.764, 3.702, and 5.082 for RBC, FFP, and PC transfusions, respectively. Third, machine learning analysis identified transfusion-related variables among the top predictors of 90-day mortality, alongside established risk factors such as age and comorbidities.

The relationship between transfusion and outcomes in ECMO patients has remained unclear despite its widespread practice^10,22–24^. While blood product support is often necessary during ECMO to manage bleeding complications and maintain adequate tissue perfusion, the optimal transfusion strategy remains undefined. This knowledge gap is particularly concerning given the high transfusion rates observed in ECMO patients compared to other critically ill populations.

Our analysis of 11,874 ECMO patients revealed several important findings. The baseline characteristics demonstrated ischemic heart disease as the predominant indication for ECMO (66.9%). The observed 90-day mortality rate of 58.8% reflects the critical nature of conditions requiring ECMO support. The non-survivor group showed significantly higher age (64.5 vs 58.0 years) and higher prevalence of all evaluated comorbidities except COVID-19. Notably, 18.5% of the total cohort had a history of cancer within the previous 5 years, reflecting the real-world practice patterns in Korea where ECMO support is considered even in patients with significant comorbidities.

Non-survivors demonstrated consistently higher normalized transfusion volumes across all blood products compared to survivors. This pattern persisted after adjusting for hospital length of stay, suggesting transfusion burden as an independent factor beyond prolonged hospitalization duration. RBC transfusion demonstrated the strongest mortality association (OR 6.764, 95% CI 6.227–7.347), followed by platelet concentrate (OR 5.082, 95% CI 4.691–5.506) and fresh frozen plasma (OR 3.702, 95% CI 3.426–4.000).

The univariable and multivariable analyses provided crucial insights into the complex relationship between transfusion and mortality. After adjusting for potential confounders including age, comorbidities, and procedural factors, transfusion volumes maintained their significant association with mortality. This finding suggests that while transfusion requirements may partly reflect disease severity, they might also independently influence outcomes through mechanisms such as transfusion-related immune modulation, volume overload, or other adverse effects associated with blood product administration. Additionally, certain comorbidities such as chronic renal failure (OR 2.443) and cancer (OR 2.29) emerged as strong independent predictors of mortality.

Our subgroup analyses across different procedural contexts further strengthen these findings. The magnitude of association between transfusion volume and mortality was remarkably consistent across different procedural categories, with significant associations observed in both patients with ischemic heart disease undergoing coronary procedures and those undergoing cardiac valve or aortic surgery. This consistency suggests that the relationship between blood product administration and outcomes may be relatively uniform across different ECMO indications, regardless of the underlying cardiac pathology or surgical intervention. These findings enhance the generalizability of our results across different clinical scenarios requiring ECMO support and suggest that transfusion optimization strategies may be broadly applicable across various ECMO patient populations.

Machine learning analysis using different models demonstrated superior predictive performance compared to traditional statistical approaches. Both Random Forest and XGBoost models achieved excellent predictive accuracy (AUC 0.900 and 0.909, respectively), surpassing conventional logistic regression (AUC 0.891). RBC transfusion-related variables consistently ranked among the top predictors of 90-day mortality across models. The consistency in model performance and variable importance rankings supports the potential significance of transfusion volumes in predicting ECMO outcomes.

This study had several limitations. First, the use of claims data significantly restricted our access to crucial laboratory parameters. We could not assess hemoglobin levels, platelet counts, activated partial thromboplastin time, prothrombin time, or international normalized ratio. This limitation prevented quantitative evaluation of anemia severity, degree of thrombocytopenia, and extent of coagulopathy, which are critical determinants of transfusion requirements in ECMO patients. Without these laboratory values, we could not fully evaluate the appropriateness of transfusion decisions or adjust our analysis for baseline hematologic status. Furthermore, the NHIS database does not contain the detailed clinical parameters required to calculate validated critical care severity scores such as APACHE II or SOFA, which would have enabled more robust adjustment for illness severity. This is a fundamental limitation of administrative claims databases, which primarily collect information for reimbursement purposes rather than clinical research. To address this limitation, we incorporated the Charlson Comorbidity Index as a measure of overall comorbidity burden, which serves as a partial proxy for baseline patient risk and is the most commonly used severity adjustment tool in NHIS data research. We also performed extensive multivariable adjustment for available clinical factors that influence both transfusion requirements and mortality, including age, comorbidities, primary diagnoses, and concurrent procedures. Nevertheless, the association between higher transfusion volumes and mortality should be interpreted with caution, as transfusion requirements likely reflect, at least in part, greater disease severity rather than direct causation of mortality. Second, the association between higher transfusion volumes and mortality requires careful interpretation regarding causality. Higher transfusion requirements likely reflect greater disease severity, coagulopathy, or bleeding complications rather than direct causation of mortality. Severely ill patients typically require more transfusions, making it challenging to determine whether increased transfusion volumes independently contribute to mortality or simply serve as a marker of illness severity. Prospective randomized controlled trials comparing different transfusion strategies in ECMO patients would help establish causality and optimal transfusion protocols. Third, the specific indications for transfusion were not available in the database, preventing analysis of transfusion appropriateness. Fourth, and importantly, we could not differentiate between veno-venous (VV) and veno-arterial (VA) ECMO modes because the Korean National Health Insurance claims data does not distinguish between these configurations. However, the predominant indication being ischemic heart disease (66.9%) suggests that VA ECMO was likely the primary mode of support in our cohort, as cardiovascular indications typically require VA ECMO support. Nevertheless, this limitation remains significant as VV and VA ECMO differ substantially in their indications, complications, and potentially in their transfusion requirements. Furthermore, we cannot distinguish between patients who received heparin-coated versus non-coated circuits, which may have different anticoagulation requirements and consequently different bleeding risk profiles. Similarly, the NHIS database does not allow differentiation between peripheral and central cannulation approaches. This is a significant limitation as central ECMO cannulation typically carries higher bleeding risk due to the more invasive nature of the procedure, potentially necessitating increased transfusion support. Despite these limitations, our large sample size provides valuable population-level insights into transfusion patterns and outcomes in ECMO patients. Another limitation of our study is the imbalance in patient distribution across centers of different volumes. As shown in Table 1, approximately 88% of ECMO procedures in our cohort were performed at large centers, while only 12% were performed at smaller centers. This substantial disproportion creates challenges for meaningful statistical comparison of outcomes between centers of different volumes. In our univariable analysis (Table 4), we did observe that patients treated at the largest centers (Q4) had better outcomes with an odds ratio of 0.64, suggesting a protective effect of high-volume centers. However, we did not include this variable in our final multivariable model due to the significant imbalance in the distribution of patients, which would result in findings predominantly driven by large-center data. Consequently, our results may be most generalizable to high-volume ECMO centers rather than smaller programs. Future research with more balanced representation across center volumes would be valuable to further explore the interactions between center volume, transfusion practices, and ECMO outcomes. Additionally, our use of proxy variables to address care heterogeneity has inherent limitations. While we included “capital area” and “ECMO center volume quartiles” to partially account for regional and institutional variability, these broad categorizations cannot fully capture the complex differences in care quality, protocols, and expertise across institutions. The capital area variable, though reflecting resource concentration, does not account for variations in ECMO management protocols, team experience, or patient selection criteria that may differ even among tertiary centers within the same region. Similarly, annual case volume quartiles, while providing some indication of institutional experience, do not necessarily reflect the quality of care, availability of specialized personnel, or adherence to best practices. More granular institutional-level data, including specific ECMO protocols, staffing patterns, and quality metrics, would be needed to more accurately assess the impact of care heterogeneity on outcomes. Another limitation of our approach is the normalization of transfusion volumes by hospital stay rather than ECMO duration. This methodological choice was primarily due to constraints in the NHIS database, which records total blood product administration during hospitalization without providing granular daily transfusion data that would allow precise allocation of transfusions to the ECMO versus post-ECMO period. Ideally, normalization by ECMO duration might provide more specific insight into transfusion requirements directly attributable to ECMO support. However, it is also important to note that ECMO-associated coagulopathy and bleeding complications often persist beyond ECMO discontinuation due to residual effects of systemic anticoagulation, platelet dysfunction, and endothelial injury. Consequently, many transfusions administered shortly after ECMO decannulation remain mechanistically linked to ECMO-induced hemostatic derangements, suggesting that total hospitalization transfusion burden remains a clinically relevant metric. Furthermore, normalization by hospital stay fails to account for the critical early-phase transfusion requirements during ECMO initiation. The initial priming of the ECMO circuit requires significant volume (typically 1–2 L), leading to immediate hemodilution that often necessitates transfusion support within the first hours of ECMO deployment. This acute hemodilution effect is most pronounced during the initiation phase when patients are already hemodynamically unstable. By normalizing transfusion volumes across the entire hospital stay, our methodology may underestimate the true transfusion burden during this most critical phase of ECMO support, potentially diluting the observed association between early transfusion intensity and outcomes.

It is important to note that certain interventions, including coronary artery bypass grafting, lung transplantation, and heart transplantation, demonstrated apparent protective effects in our analysis. However, this finding warrants careful interpretation. Rather than suggesting a direct protective effect of these procedures, these associations likely reflect important differences in patient selection, clinical context, and management strategies. Patients undergoing transplantation typically have ECMO planned as part of their perioperative management strategy, undergo thorough pre-procedural evaluation, and receive care in specialized centers with established protocols. Similarly, patients selected for CABG while on ECMO support may represent a subset with more favorable anatomy and hemodynamic stability compared to those managed conservatively or with percutaneous intervention. This represents an important selection bias, as these planned or semi-elective ECMO cases fundamentally differ from emergency ECMO deployment scenarios such as out-of-hospital cardiac arrest or acute decompensated heart failure. The lower mortality observed in these subgroups likely reflects differences in baseline risk profiles, the elective nature of ECMO institution, and potentially more optimized management pathways rather than a direct protective effect of the procedures themselves. Future studies should stratify outcomes by the elective versus emergent nature of ECMO deployment to better understand these associations and develop tailored management strategies for different ECMO indications.

Despite these limitations, the use of nationwide real-world data encompassing nearly the entire Korean population provided robust evidence regarding transfusion patterns and outcomes in ECMO patients. While most ECMO studies are limited by small sample sizes due to the specialized nature of the intervention, our analysis of over 11,000 ECMO cases represents one of the largest cohorts in the literature, offering valuable insights that may not be obtainable from single-center studies or smaller cohorts.

Importantly, our study suggests that restrictive transfusion strategies may demonstrate non-inferiority regarding mortality rates compared to liberal transfusion approaches in ECMO patients, which provides crucial clinical context for interpreting our findings. The strong association we observed between higher normalized transfusion volumes and increased mortality risk aligns with emerging evidence supporting more conservative transfusion practices in critically ill patients. These findings suggest that clinicians may safely adopt lower transfusion thresholds without compromising patient survival, potentially reducing both transfusion-related complications and healthcare utilization while maintaining optimal patient outcomes.

## Conclusions

In conclusion, blood transfusion volume normalized by hospital stay is a modifiable factor with significant implications for ECMO patient outcomes. Strategies to optimize transfusion practices could improve survival rates and patient safety.

## Electronic supplementary material

Below is the link to the electronic supplementary material.

Supplementary Material 1

## Data Availability

The datasets used and/or analyzed during the current study are available from the corresponding author on reasonable request.

## References

[1] Shekar, K. et al. Extracorporeal life support devices and strategies for management of acute cardiorespiratory failure in adult patients: A comprehensive review. *Crit. Care* **18**, 219 (2014). 25032748 10.1186/cc13865 PMC4057103

[2] Gerke, A. K., Tang, F., Cavanaugh, J. E., Doerschug, K. C. & Polgreen, P. M. Increased trend in extracorporeal membrane oxygenation use by adults in the United States since 2007. *BMC Res. Notes.* **8**, 686 (2015). 26581610 10.1186/s13104-015-1678-7 PMC4650500

[3] Raasveld, S. J. et al. Transfusion of red blood cells in venoarterial extracorporeal membrane oxygenation: A multicenter retrospective observational cohort study. *Transfusion* **63**, 1809–1820 (2023). 37668074 10.1111/trf.17505

[4] Jiritano, F. et al. Platelets and extra-corporeal membrane oxygenation in adult patients: a systematic review and meta-analysis. *Intensive Care Med.* **46**, 1154–1169 (2020). 32328725 10.1007/s00134-020-06031-4 PMC7292815

[5] Raasveld, S. J. et al. Red blood cell transfusion in the intensive care unit. *JAMA* **330**, 1852–1861 (2023). 37824112 10.1001/jama.2023.20737 PMC10570913

[6] Tigano, S., Sanfilippo, F., Capuano, P., Arcadipane, A. & Martucci, G. Current practice optimization suggestions and future perspectives on transfusion in patients supported by extracorporeal membrane oxygenation: A narrative review. *Ann. Blood.* **9**, 9 (2023).

[7] Carson, J. L. et al. Red blood cell transfusion: 2023 AABB international guidelines. *JAMA* **330**, 1892–1902 (2023). 37824153 10.1001/jama.2023.12914

[8] Ramanathan, K. et al. Blood transfusion during extracorporeal membrane oxygenation: An ELSO position statement. *ASAIO J.* **70**, 719–720 (2024). 39024410 10.1097/MAT.0000000000002275

[9] Martucci, G. et al. Transfusion practice in patients receiving VV ECMO (PROTECMO): A prospective, multicentre, observational study. *Lancet Respir. Med.* **11**, 245–255 (2023). 36240836 10.1016/S2213-2600(22)00353-8

[10] Pratt, E. H. et al. Association of RBC transfusion thresholds and outcomes in medical patients with acute respiratory failure supported with extracorporeal membrane oxygenation: A single-center retrospective cohort study. *Chest* **166**, 1406–1416 (2024). 38986867 10.1016/j.chest.2024.05.043 PMC11638546

[11] Kim, Y. I. et al. Cohort profile: National health insurance service-senior (NHIS-senior) cohort in Korea. *BMJ Open* **9**, e024344 (2019). 31289051 10.1136/bmjopen-2018-024344 PMC6615810

[12] Cheol Seong, S. et al. Data resource profile: The National Health Information Database of the National Health Insurance Service in South Korea. *Int. J. Epidemiol.* **46**, 799–800 (2017). 27794523 10.1093/ije/dyw253 PMC5837262

[13] Kim, L., Kim, J. A. & Kim, S. A guide for the utilization of Health Insurance Review and Assessment Service National Patient Samples. *Epidemiol Health.* **36**, e2014008 (2014). 25078381 10.4178/epih/e2014008 PMC4151963

[14] Salman, H. A., Kalakech, A. & Steiti, A. Random forest algorithm overview. *Babylon. J. Mach. Learn.* **2024**, 69–79 (2024).

[15] Dinç, S. & Aygün, R. S. Evaluation of hyperspectral image classification using random forest and Fukunaga–Koontz transform. In *Paper Presented at: Machine Learning and Data Mining in Pattern Recognition* (Berlin, 2013).

[16] Lee, K. S. & Ham, B. J. Machine learning on early diagnosis of depression. *Psychiatry Investig.* **19**, 597–605 (2022). 36059048 10.30773/pi.2022.0075 PMC9441463

[17] Lee, K. S. & Kim, E. S. Explainable artificial intelligence in the early diagnosis of gastrointestinal disease. *Diagnostics (Basel)* **12**, 2740 (2022). 36359583 10.3390/diagnostics12112740 PMC9689865

[18] Agarwal, A., Kenney, A. M., Tan, Y. S., Tang, T. M. & Yu, B. MDI+: A flexible random forest-based variable importance framework. arXiv preprint https://arxiv.org/abs/2307.01932 (2023).

[19] Scornet, E. Trees, forests, and impurity-based variable importance in regression. *Annales de l’Institut Henri Poincaré, Probabilités et Statistiques.* **59**(21–52), 32 (2023).

[20] Breiman, L. Random forests. *Mach. Learn.* **45**, 5–32 (2001).

[21] Lee, K. S. & Ahn, K. H. Application of artificial intelligence in early diagnosis of spontaneous preterm labor and birth. *Diagnostics (Basel)* **10**, 733 (2020). 32971981 10.3390/diagnostics10090733 PMC7555184

[22] Qin, C. X. et al. Blood utilization and clinical outcomes in extracorporeal membrane oxygenation patients. *Anesth. Analg.* **131**, 901–908 (2020). 32304461 10.1213/ANE.0000000000004807 PMC7853404

[23] Ng, P. Y. et al. Restrictive and liberal transfusion strategies in extracorporeal membrane oxygenation: A retrospective observational study. *Transfusion* **63**, 294–304 (2023). 36511445 10.1111/trf.17221

[24] Raasveld, S. J. et al. RBC transfusion in venovenous extracorporeal membrane oxygenation: A multicenter cohort study. *Crit. Care Med.* **50**, 224–234 (2022). 35100195 10.1097/CCM.0000000000005398

