# Supplementary material for: Association between blood transfusion and early mortality in patient undergoing extracorporeal membrane oxygenation
Source: Sci Rep. 2025 Jul 25;15:27145. doi: 10.1038/s41598-025-11702-7 (PMC12297166; doi:10.1038/s41598-025-11702-7)
Supplement: Supplementary file 1 — Supplementary Material 1 [file 41598_2025_11702_MOESM1_ESM.docx]

**Supplementary Table 1. Univariable and multivariable analysis of logistic regression for the 90-day death (ischemic heart disease with percutaneous coronary intervention subgroup, N=2,110)**

| **Variables** | **Univariable** |  | **Multivariable** |  |
| --- | --- | --- | --- | --- |
|  | **OR (95% CI)** | **P value** | **OR (95% CI)** | **P value** |
| Female | 1.07 (0.88–1.30) | 0.477 | 0.98 (0.75–1.29) | 0.908 |
| Age | 1.03 (1.02–1.03) | <0.001 | 1.04 (1.03–1.05) | <0.001 |
| Capital area | 0.83 (0.69–1.00) | 0.044 | 0.81 (0.63–1.03) | 0.081 |
| Hypertension | 1.37 (1.14–1.64) | 0.001 | 1.12 (0.82–1.51) | 0.481 |
| Diabetes Mellitus | 1.16 (0.97–1.39) | 0.098 | 1.30 (0.97–1.73) | 0.075 |
| Dyslipidemia | 1.08 (0.90–1.30) | 0.417 | 0.94 (0.70–1.28) | 0.700 |
| Ischemic stroke | 1.25 (0.93–1.67) | 0.133 | 1.17 (0.78–1.77) | 0.447 |
| Myocardial Infarction | 0.87 (0.70–1.09) | 0.229 | 0.72 (0.51–1.00) | 0.050 |
| Congestive Heart Failure | 1.02 (0.75–1.39) | 0.903 | 0.88 (0.57–1.36) | 0.571 |
| Cancer | 1.28 (1.00–1.64) | 0.053 | 1.67 (1.17–2.38) | 0.004 |
| Chronic Kidney Disease | 1.47 (1.12–1.91) | 0.005 | 1.84 (1.25–2.70) | 0.002 |
| COVID19 | 0.43 (0.13–1.42) | 0.166 | 0.45 (0.11–1.85) | 0.270 |
| Charlson Comorbidity Index | 0.82 (0.79–0.84) | <0.001 | 0.76 (0.73–0.79) | <0.001 |
| Diagnosis at ECMO |  |  |  |  |
| Ischemic Heart Disease | 0.91 (0.76–1.09) | 0.301 | 0.97 (0.57–1.66) | 0.918 |
| STEMI | 0.87 (0.70–1.09) | 0.236 | 1.03 (0.71–1.50) | 0.870 |
| NSTEMI | 0.92 (0.70–1.22) | 0.580 | 0.97 (0.64–1.49) | 0.898 |
| Angina | 0.94 (0.77–1.17) | 0.596 | 0.99 (0.70–1.40) | 0.955 |
| PCI | 1.00 (0.83–1.20) | 0.963 | 1.13 (0.72–1.79) | 0.594 |
| Coronary Artery Bypass Grafting | 0.59 (0.42–0.82) | 0.002 | 0.35 (0.20–0.62) | <0.001 |
| Myocarditis | 0.38 (0.26–0.54) | <0.001 | 0.51 (0.31–0.83) | 0.007 |
| Pulmonary thromboembolism | 0.62 (0.39–0.99) | 0.046 | 0.36 (0.18–0.73) | 0.004 |
| Cardiomyopathy | 0.61 (0.45–0.82) | 0.001 | 0.87 (0.57–1.33) | 0.528 |
| Heart failure | 0.88 (0.71–1.09) | 0.229 | 1.14 (0.85–1.51) | 0.379 |
| Valve surgery | 1.57 (1.01–2.46) | 0.046 | 0.57 (0.30–1.10) | 0.093 |
| Cardiac arrest | 1.17 (0.93–1.48) | 0.178 | 0.95 (0.68–1.32) | 0.753 |
| Pulmonary disease | 1.23 (1.02–1.48) | 0.032 | 1.09 (0.83–1.42) | 0.542 |
| Aorta surgery | 1.58 (0.86–2.91) | 0.144 | 0.57 (0.19–1.67) | 0.306 |
| Ventricular assist device | 0.07 (0.01–0.60) | 0.015 | 0.11 (0.01–1.16) | 0.066 |
| ECMO duration, day | 1.00 (0.98–1.01) | 0.438 | 1.01 (0.99–1.03) | 0.163 |
| RBC/hospital stay | 5.26 (4.26–6.48) | <0.001 | 3.43 (2.58–4.57) | <0.001 |
| PC/hospital stay | 1.85 (1.70–2.01) | <0.001 | 1.33 (1.20–1.47) | <0.001 |
| FFP/hospital stay | 3.82 (3.09–4.73) | <0.001 | 1.47 (1.15–1.89) | 0.002 |
| CPR at ER | 1.31 (1.03–1.67) | 0.031 | 1.07 (0.75–1.52) | 0.726 |

CPR, cardiopulmonary resuscitation; ECMO, extracorporeal membrane oxygenation; FFP, fresh frozen plasma; NSTEMI, non-ST elevation myocardial infarction; PC, platelet concentrates, PCI, percutaneous coronary intervention; RBC, red blood cell; STEMI, ST elevation myocardial infarction.

**Supplementary Table 2. Univariable and multivariable analysis of logistic regression for the 90-day death (heart valve or aorta surgery subgroup, N=1,024)**

| **Variables** | **Univariable** |  | **Multivariable** |  |
| --- | --- | --- | --- | --- |
|  | **OR (95% CI)** | **P value** | **OR (95% CI)** | **P value** |
| Female | 1.44 (1.11–1.87) | 0.006 | 1.13 (0.78–1.63) | 0.531 |
| Age | 1.04 (1.03–1.05) | <0.001 | 1.03 (1.02–1.05) | <0.001 |
| Capital area | 1.00 (0.77–1.31) | 0.970 | 1.05 (0.72–1.51) | 0.811 |
| Hypertension | 1.73 (1.26–2.37) | 0.001 | 1.35 (0.82–2.22) | 0.242 |
| Diabetes Mellitus | 2.07 (1.59–2.69) | <0.001 | 2.51 (1.68–3.76) | <0.001 |
| Dyslipidemia | 1.39 (1.04–1.86) | 0.027 | 0.93 (0.59–1.47) | 0.755 |
| Ischemic stroke | 1.08 (0.76–1.54) | 0.677 | 0.82 (0.49–1.36) | 0.438 |
| Myocardial Infarction | 1.29 (0.96–1.73) | 0.086 | 0.95 (0.61–1.48) | 0.826 |
| Congestive Heart Failure | 1.47 (1.09–1.99) | 0.011 | 1.01 (0.66–1.57) | 0.949 |
| Cancer | 1.22 (0.83–1.78) | 0.309 | 2.31 (1.29–4.12) | 0.005 |
| Chronic Kidney Disease | 1.92 (1.37–2.70) | <0.001 | 2.16 (1.33–3.49) | 0.002 |
| COVID19 | 0.53 (0.03–8.53) | 0.656 | 0.77 (0.01–70.93) | 0.912 |
| Charlson Comorbidity Index | 0.84 (0.81–0.88) | <0.001 | 0.75 (0.70–0.80) | <0.001 |
| Diagnosis at ECMO |  |  |  |  |
| Ischemic Heart Disease | 0.85 (0.65–1.10) | 0.211 | 0.53 (0.23–1.21) | 0.132 |
| STEMI | 0.71 (0.29–1.69) | 0.434 | 0.51 (0.10–2.70) | 0.430 |
| NSTEMI | 0.76 (0.45–1.26) | 0.280 | 0.86 (0.39–1.86) | 0.694 |
| Angina | 0.93 (0.69–1.26) | 0.652 | 1.65 (0.87–3.13) | 0.124 |
| PCI | 0.81 (0.48–1.35) | 0.415 | 1.75 (0.75–4.07) | 0.194 |
| Coronary Artery Bypass Grafting | 0.94 (0.71–1.24) | 0.656 | 1.91 (0.96–3.79) | 0.067 |
| Myocarditis | 0.01 (0.00–INF) | 0.999 | 0.01 (0.00–INF) | 0.997 |
| Pulmonary thromboembolism | 0.60 (0.22–1.68) | 0.334 | 1.55 (0.40–5.98) | 0.522 |
| Cardiomyopathy | 0.69 (0.41–1.14) | 0.148 | 0.70 (0.32–1.54) | 0.378 |
| Heart failure | 1.38 (1.04–1.84) | 0.028 | 1.42 (0.94–2.12) | 0.094 |
| Valve surgery | 1.88 (1.65–2.13) | <0.001 |  |  |
| Cardiogenic shock | 1.66 (1.05–2.62) | 0.030 | 1.51 (0.79–2.87) | 0.210 |
| Cardiac arrest | 1.10 (0.62–1.96) | 0.744 | 0.95 (0.37–2.41) | 0.915 |
| Pulmonary disease | 0.88 (0.65–1.19) | 0.414 | 1.22 (0.81–1.85) | 0.346 |
| Aorta surgery | 0.88 (0.65–1.20) | 0.416 | 0.94 (0.55–1.62) | 0.836 |
| Ventricular assist device | 0.21 (0.04–1.09) | 0.064 | 0.17 (0.02–1.78) | 0.138 |
| ECMO duration, day | 1.00 (0.97–1.02) | 0.640 | 1.03 (1.00–1.06) | 0.094 |
| RBC/hospital stay | 3.41 (2.76–4.22) | <0.001 | 1.85 (1.41–2.43) | <0.001 |
| PC/hospital stay | 1.95 (1.75–2.19) | <0.001 | 1.47 (1.28–1.69) | <0.001 |
| FFP/hospital stay | 4.33 (3.30–5.67) | <0.001 | 1.71 (1.20–2.44) | 0.003 |
| CPR at ER | 1.48 (0.65–3.36) | 0.347 | 1.18 (0.31–4.49) | 0.804 |

CPR, cardiopulmonary resuscitation; ECMO, extracorporeal membrane oxygenation; FFP, fresh frozen plasma; NSTEMI, non-ST elevation myocardial infarction; PC, platelet concentrates, PCI, percutaneous coronary intervention; RBC, red blood cell; STEMI, ST elevation myocardial infarction.

**Supplementary Table 3. Feature importance of Random Forest, XGboost and Logistic Regression**

| Model | **Random Forest** | | **XGBoost** | | **Logistic Regression** | |
| --- | --- | --- | --- | --- | --- | --- |
|  | Feature | Importance | Feature | Importance | Feature | Importance |
| 1 | RBC/hospital days | 0.183 | Charlson comorbidity index | 0.157 | Ventricular assist device | 1.271 |
| 2 | PC/hospital days | 0.139 | Age | 0.134 | RBC/hospital days | 0.866 |
| 3 | FFP/hospital days | 0.110 | RBC/hospital days | 0.130 | Chronic kidney disease | 0.771 |
| 4 | Charlson comorbidity index | 0.106 | PC/hospital days | 0.099 | CABG | 0.740 |
| 5 | Age | 0.092 | FFP/hospital days | 0.084 | Valve surgery | 0.604 |
| 6 | ECMO operation day | 0.060 | ECMO operation day | 0.081 | Cancer | 0.570 |
| 7 | Economic status | 0.053 | Cancer | 0.037 | Aorta surgery | 0.563 |
| 8 | Capital area | 0.013 | Chronic kidney disease | 0.030 | Pulmonary thromboembolism at ECMO | 0.562 |
| 9 | Pulmonary disease | 0.013 | Diabetes mellitus | 0.029 | Myocarditis at ECMO | 0.438 |
| 10 | Female | 0.013 | CABG | 0.022 | PC/hospital days | 0.385 |
| 11 | Diabetes mellitus | 0.013 | Pulmonary disease | 0.019 | Diabetes mellitus | 0.365 |
| 12 | Hypertension | 0.012 | Valve surgery | 0.016 | Cariogenic shock at ECMO | 0.285 |
| 13 | Cancer | 0.012 | Ischemic stroke | 0.014 | Ischemic stroke | 0.274 |
| 14 | Dyslipidemia | 0.011 | Cardiomyopathy at ECMO | 0.014 | Charlson comorbidity index | 0.264 |
| 15 | Heart failure at ECMO | 0.011 | Female | 0.012 | NSTEMI | 0.238 |
| 16 | Chronic kidney disease | 0.010 | Pulmonary thromboembolism at ECMO | 0.010 | FFP/hospital days | 0.236 |
| 17 | Cardiac arrest at ECMO | 0.010 | Aorta surgery | 0.010 | Heart failure at ECMO | 0.227 |
| 18 | Cariogenic shock at ECMO | 0.010 | Cariogenic shock at ECMO | 0.007 | Angina | 0.202 |
| 19 | Myocardial infarction | 0.010 | Myocarditis at ECMO | 0.007 | COVID-19 | 0.188 |
| 20 | PCI | 0.009 | Hypertension | 0.006 | Pulmonary disease | 0.149 |
| 21 | Ischemic heart disease | 0.009 | Ventricular assist device | 0.006 | Not categorized | 0.140 |
| 22 | CABG | 0.008 | Economic status | 0.004 | Female | 0.120 |
| 23 | CPR at ER | 0.008 | Dyslipidemia | 0.004 | Myocardial infarction | 0.113 |
| 24 | Angina | 0.008 | Heart failure at ECMO | 0.004 | STEMI | 0.111 |
| 25 | Ischemic stroke | 0.008 | CPR at ER | 0.004 | Ischemic heart disease | 0.107 |
| 26 | Valve surgery | 0.007 | Cardiac arrest at ECMO | 0.003 | PCI | 0.100 |
| 27 | Congestive heart failure | 0.007 | PCI | 0.003 | Hypertension | 0.089 |
| 28 | Cardiomyopathy at ECMO | 0.007 | Angina | 0.003 | Capital area | 0.084 |
| 29 | STEMI | 0.006 | STEMI | 0.003 | Congestive heart failure | 0.075 |
| 30 | Not categorized | 0.006 | Congestive heart failure | 0.001 | CPR at ER | 0.063 |
| 31 | Pulmonary thromboembolism at ECMO | 0.006 | Not categorized | 0.001 | Dyslipidemia | 0.058 |
| 32 | NSTEMI | 0.006 | Capital area | 0.000 | Cardiac arrest at ECMO | 0.058 |
| 33 | Myocarditis at ECMO | 0.005 | Myocardial infarction | 0.000 | Age | 0.037 |
| 34 | Aorta surgery | 0.004 | Ischemic heart disease | 0.000 | Cardiomyopathy at ECMO | 0.026 |
| 35 | COVID-19 | 0.002 | NSTEMI | 0.000 | ECMO operation day | 0.009 |
| 36 | Ventricular assist device | 0.001 | COVID-19 | 0.000 | Economic status | 0.007 |

* Abbreviations: CABG (coronary artery bypass grafting), CCI (Charlson comorbidity index), CPR (cardiopulmonary resuscitation), ECMO (extracorporeal membrane oxygenation), FFP (fresh frozen plasma), IHD (ischemic heart disease), NSTEMI (non-ST-elevation myocardial infarction), PC (platelet concentrate), PCI (percutaneous coronary intervention), RBC (red blood cell), STEMI (ST-elevation myocardial infarction).

* IHD + coronary procedure: Includes patients diagnosed with IHD or those not diagnosed with IHD but who underwent PCI or CABG.

* Not categorized: Refers to cases where the diagnosis codes are not classified under IHD, cardiogenic shock, cardiac arrest, pulmonary embolism, or pulmonary disease.
